# Causal role of immune cells in diabetic nephropathy: a bidirectional Mendelian randomization study

**DOI:** 10.3389/fendo.2024.1357642

**Published:** 2024-09-13

**Authors:** Shang-Yuan Wang, Yang Yu, Xiao-Li Ge, Shuming Pan

**Affiliations:** Department of Emergency Medicine, Xinhua Hospital, School of Medicine, Shanghai Jiao Tong University, Shanghai, China

**Keywords:** diabetic nephropathy, Mendelian randomization, immune cells, HLA-DR, monocyte, dendritic cells

## Abstract

**Background:**

Diabetic nephropathy (DN) stands as a pervasive chronic renal disease worldwide, emerging as the leading cause of renal failure in end-stage renal disease. Our objective is to pinpoint potential immune biomarkers and evaluate the causal effects of prospective therapeutic targets in the context of DN.

**Methods:**

We employed Mendelian randomization (MR) analysis to examine the causal associations between 731 immune cell signatures and the risk of DN. Various analytical methods, including inverse-variance weighted (IVW), MR-Egger, weighted median, simple mode, and weighted mode, were employed for the analysis. The primary analytical approach utilized was the inverse-variance weighted (IVW) method. To ensure the reliability of our findings, we conducted comprehensive sensitivity analyses to assess the robustness, heterogeneity, and presence of horizontal pleiotropy in the results. Statistical powers were also calculated. Ultimately, a reverse Mendelian randomization (MR) analysis was conducted to assess the potential for reverse causation.

**Results:**

After Benjamini & Hochberg (BH) correction, four immunophenotypes were identified to be significantly associated with DN risk: HLA DR on Dendritic Cell (OR=1.4460, 95% CI = 1.2904~1.6205, P=2.18×10^−10^, P.adjusted= 1.6×10^−7^), HLA DR on CD14^+^ CD16^−^ monocyte (OR=1.2396, 95% CI=1.1315~1.3580, P=3.93×10^−6^, P.adjusted = 0.00143). HLA DR on CD14^+^ monocyte (OR=1.2411, 95% CI=1.12957~1.3637, P=6.97×10^−6^, P.adjusted=0.0016), HLA DR on plasmacytoid Dendritic Cell (OR=1.2733, 95% CI= 1.1273~1.4382, P= 0.0001, P.adjusted = 0.0183). Significant heterogeneity of instrumental variables was found in the four exposures, and significant horizontal pleiotropy was only found in HLA DR on Dendritic Cell. The bidirectional effects between the immune cells and DN were not supported.

**Conclusion:**

Our research illustrated the intimate association between immune cells and DN, which may contribute to a deeper understanding of the intricate mechanisms underlying DN and aid in the identification of novel intervention target pathways.

## Introduction

One of the most frequent complications in people with diabetes mellitus (DM) is DN, which is a major global cause of end-stage renal disease (ESRD) (1). Worldwide, DN affects between 25% and 40% of DM patients (2). An estimated glomerular filtration rate (eGFR) of less than 60 ml/min/1.73 m^2^ and a urine albumin-to-creatinine ratio more than 30 mg/g are two indicators of persistent albuminuria, which is often necessary for the diagnosis of diabetic kidney disease (DN) (3). The present treatment options for DN are limited, despite its high prevalence and notable impact on morbidity and mortality (4). Finding new biomarkers for the early diagnosis of DN and promising pharmacological targets for the treatment of DN is therefore imperative.

It was formerly believed that DN was caused by high blood sugar levels producing non-immunological, metabolic, or hemodynamic damage in the kidneys (1). However, current research indicates that DN is an inflammatory illness, and immune cells from both innate and adaptive immunity, including macrophages and T cells, may contribute to the illness’s exacerbation (5). In the diabetic environment, hyperglycemia (6), advanced glycation end-products (AGEs) (7), angiotensin II (8), and oxidative stress (9) trigger numerous signaling cascades that initiate the recruitment and activation of immune cells. This, in turn, facilitates the development of inflammation, ultimately resulting in a range of pathological alterations in DN (10). A study on the autopsy of DN found that the accumulation of macrophages in the kidneys suggested a decline in kidney function (11). Mechanistically speaking, hyperglycemia triggers an elevation in the expression of chemokines and adhesion molecules, effectively enhancing the recruitment of monocytes to the kidney (12). Human monocytes can be categorized into three distinct subpopulations: the classical type (CD14^+^CD16^−^), the non-classical type (CD14^dim^CD16^+^), and the intermediate type (CD14^+^CD16^+^) (13). Subsequently, these monocytes undergo maturation into macrophages and secrete inflammatory cytokines, further amplifying the inflammatory response. A study has revealed that T cells also migrate to the kidney of individuals with diabetes and significantly contribute to the advancement of DN (14). A recent study (15) has revealed that the ratio of monocytes to lymphocytes serves as a significant indicator for predicting the occurrence of DN among individuals suffering from type 2 diabetes. Dendritic cells are crucial antigen-presenting cells in the immune system (16). They present antigens to different receptors on various immune cells, thereby activating both innate and adaptive immune responses (17). Although dendritic cells have been observed to infiltrate and accumulate in diabetes nephropathy, the role of dendritic cells in DN is still poorly studied (18). Increasing evidence suggests that kidney dendritic cells are involved in renal injury in DN, and their activation within the kidney may be a pivotal factor in disease progression (19).The identification of inflammatory markers holds immense potential in enhancing the diagnosis and treatment of DN (20).

Mendelian randomization study is a statistical method that can unveil causal relationships (21). Specifically, Mendelian randomization is based on whole-genome sequencing data GWAS data, using single nucleotide polymorphisms (SNPs) as instrumental variables (IVs) to reveal causal relationships. Its principle mainly includes the following points. (i) The instrumental variable is independent of the confounding factor. (ii) The instrumental variable is associated with the exposure factor. (iii) The instrumental variable is not associated with the outcome variable, and it can only be associated with the outcome variable through the exposure factor to exclude restrictive criteria (22). The random assignment of these genetic variants in a population is independent of confounding factors, so Mendelian randomization can mimic clinical randomized controlled experiments (23). Researching the causal association between immune cells and DN could lead to novel discoveries for mechanistic studies, targeted therapies, and tracking the development of DN.

## Results

### Exploration of the causal effect of immunophenotypes on DN

**Figure 1:**
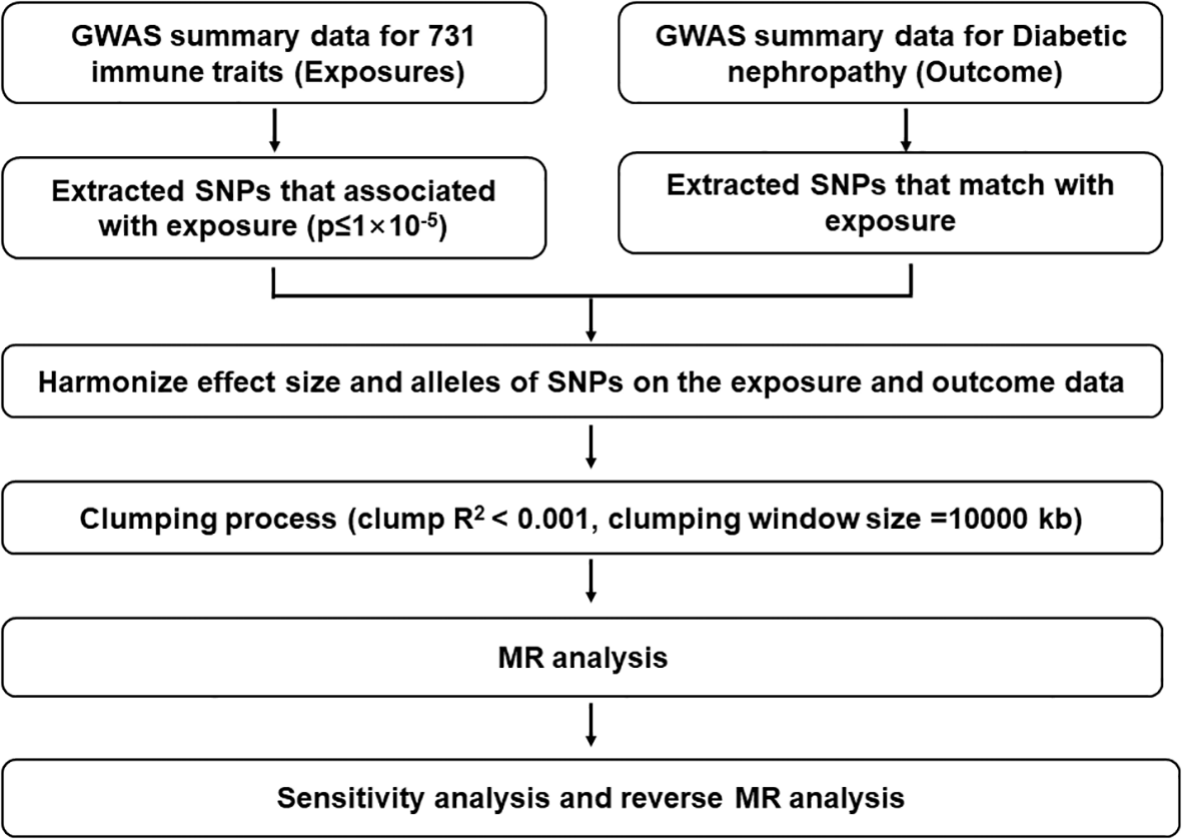
Description of the study design in this bidirectional MR study. The whole workflow of MR analysis. LD, linkage disequilibrium; MR, Mendelian randomization; SNPs, single‐nucleotide polymorphisms.

**Figure 2:**
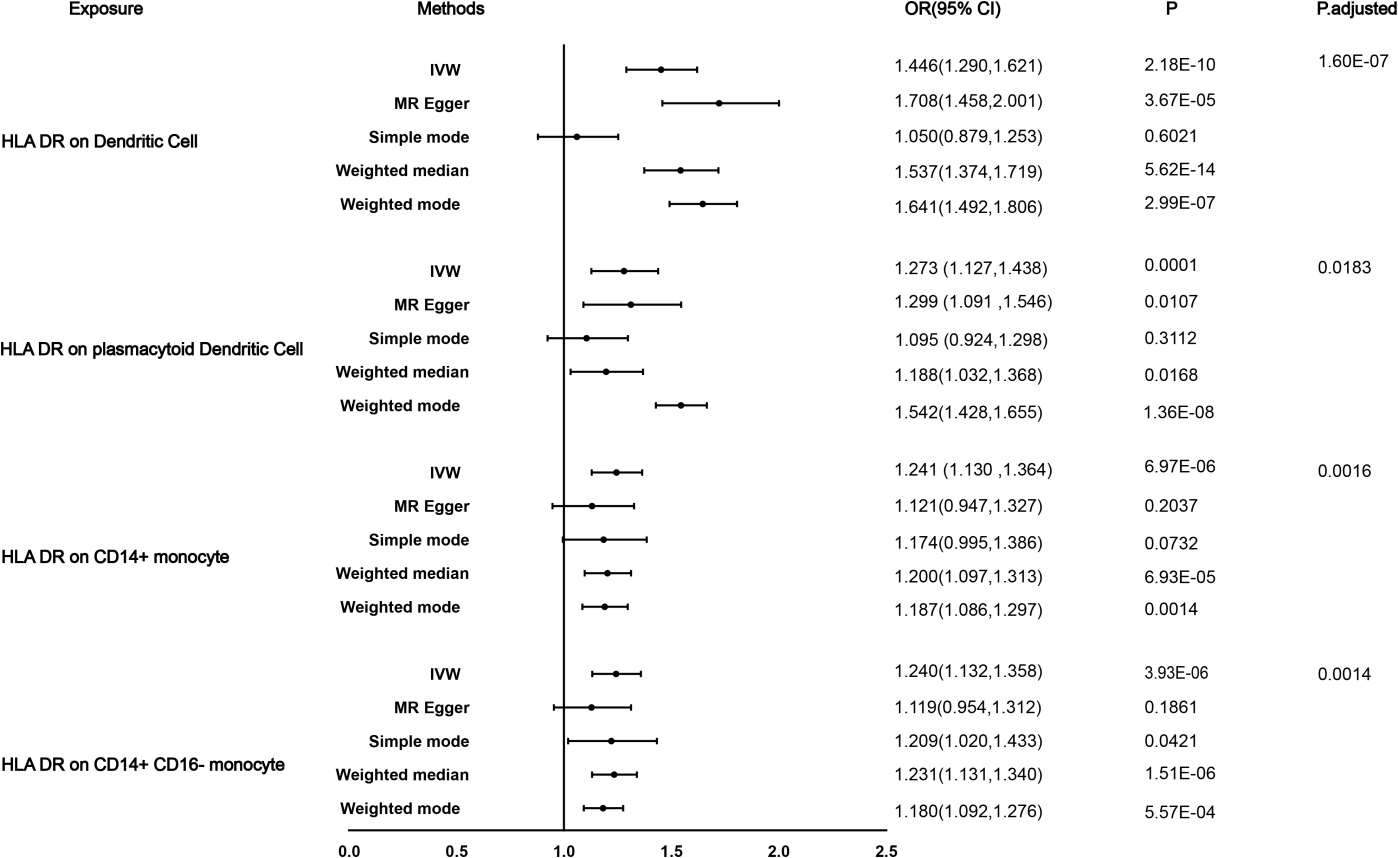
Causal effects for immune traits on DN susceptibility. Summary of the Mendelian randomization (MR) estimates derived from the inverse-variance weighted (IVW), weighted median (WM), MR-Egger, and weighted mode, simple mode methods.

**Figure 3:**
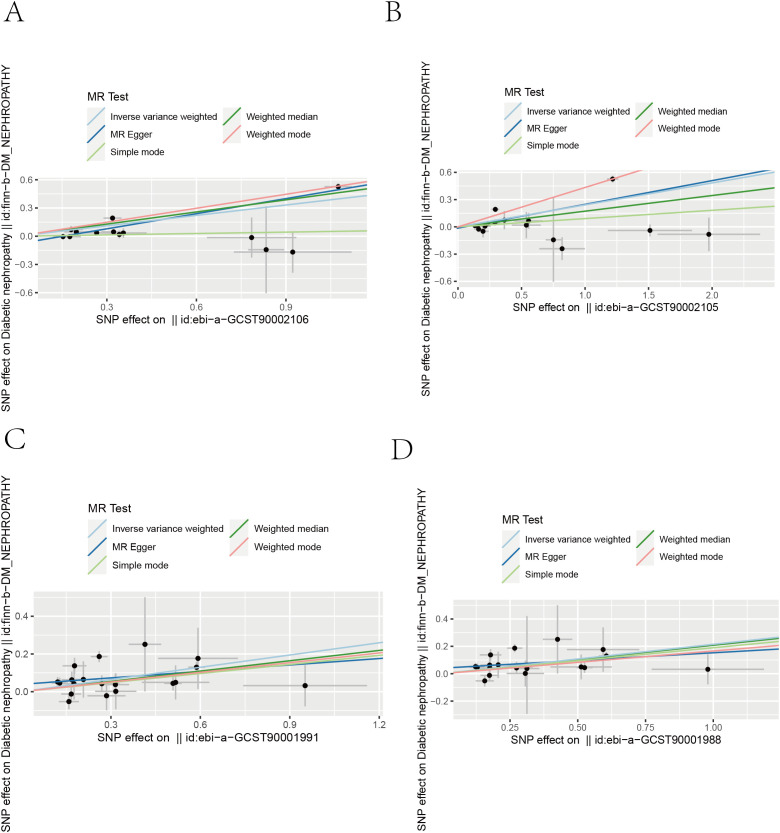
Scatter plots from genetically predicted immunophenotypes on DN. Scatter plots of the five MR tests in causal associations from four immune cell features to DN. **(A)** HLA DR on Dendritic Cell. **(B)** HLA DR on plasmacytoid Dendritic Cell **(C)** HLA DR on CD14+ monocyte. **(D)** HLA DR on CD14+ CD16− monocyte.

We performed a Mendelian randomization analysis as shown in **Figure 1**. The instrumental variables for Mendelian randomization analysis are in **Supplementary Table 1**. After BH adjustment (P.adjusted<0.05), we detected four immunophenotypes which have promoted effects on DN: HLA DR on Dendritic Cell (dendritic cell panel), HLA DR on CD14^+^ CD16^−^ monocyte (monocyte panel), HLA DR on CD14^+^ monocyte (monocyte panel), and HLA DR on plasmacytoid Dendritic Cell (dendritic cell panel). The odds ratio (OR) of HLA DR on Dendritic Cell on DN risk was estimated to be 1.4460 (95% CI= 1.2904~1.6205, P=2.18×10^−10^, P.adjusted= 1.6×10^−7^, **Figure 2**) by using the IVW method. The OR of HLA DR on CD14^+^ CD16^-^ monocyte on DN risk was estimated to be 1.2396 (95% CI=1.1315~1.3580, P=3.93×10^−6^, P.adjusted = 0.0014, **Figure 2**) by using the IVW method. The OR of HLA DR on CD14^+^ monocyte on DN risk was estimated to be 1.2411 (95% CI=1.1295~1.3637, P=6.97×10^−6^, P.adjusted=0.0016, **Figure 2**) by using the IVW method. The OR of HLA DR on plasmacytoid Dendritic Cell on DN risk was estimated to be 1.2733 (95% CI= 1.1273~1.4382, P= 0.0001, P.adjusted = 0.01835, **Figure 2**, **Supplementary Table 2**) by using the IVW method. Similar results were obtained using four additional methods (**Supplementary Table 3**). **Figure 3** shows the scatter plot for effect sizes of SNPs for immunophenotypes and those for DN.

### Sensitivity analyses

The MR-Egger intercept (P>0.05) indicated that only HLA DR on Dendritic Cell demonstrated pleiotropy in our results (**Table 1**, **Supplementary Table 4**). Furthermore, the results in **Table 1** and **Supplementary Table 5** showed that all P values of the Q test analysis were<0.05, indicating that heterogeneity existed. Importantly, as shown in the leave-one-out analysis results, no marked difference was found in causal estimations of immune cell signatures on DN, suggesting that none of the identified causal associations were driven by any single IV (**Supplementary Figure 1**). Our study provides 100% power to detect the causal effect of immune cells on DN (**Table 1**).

**Table 1:** Sensitivity analysis of the causal association between immunophenotypes and the risk of DN.

Exposure	Cochrane Q test		MR-Egger		Statistical power
	Q value	*p*	Egger_intercept	*p*	
HLA DR on dendritic cell	58.7751	3.77E−08	−0.0819	0.0261	1
HLA DR on CD14^+^ CD16^−^ monocyte	40.2525	0.0019	0.0405	0.1493	1
HLA DR on CD14^+^ monocyte	41.4289	0.0013	0.0391	0.1757	1
HLA DR on plasmacytoid Dendritic Cell	127.2844	7.22E−20	−0.0138	0.7541	1

### Exploration of the causal effect of DN onset on immunophenotypes

The reverse Mendelian randomization study did not find any causal relationship between DN and the four immune cell types we identified (**Table 2**). Furthermore, we used DN as the exposure and 731 immune cell types as the outcome, and the results showed that none of the p-values passed multiple testing corrections (**Supplementary Table 6**).

**Table 2:** Causal effects for DN on immunophenotypes.

Exposure	Outcome	IVW	
		OR (95% CI)	*p*
Diabetic nephropathy	HLA DR on Dendritic Cell	1.1964(0.9600,1.4911)	0.1102
Diabetic nephropathy	HLA DR on CD14^+^ CD16^−^ monocyte	1.1279(0.8034,1.5834)	0.4865
Diabetic nephropathy	HLA DR on CD14^+^ monocyte	1.1204(0.8063,1.5568)	0.4981
Diabetic nephropathy	HLA DR on plasmacytoid Dendritic Cell	1.1849(0.9524,1.4740)	0.1277

## Discussion

This study marks a pioneering use of Mendelian randomization to scrutinize the causal connection between immune cell characteristics and the susceptibility to DN. The findings indicate that only four distinct immune cell types exhibit a causal association with the risk of DN.

Monocytes play a pivotal role in the pathogenesis of DN (24). Heightened infiltration of monocytes into renal tissue has been documented in patients with DN, where these cells contribute to the release of pro-inflammatory cytokines, including tumor necrosis factor-alpha (TNF-α) and interleukin-1 beta (IL-1β), thereby initiating renal inflammation and subsequent tissue damage. Moreover, monocytes have the ability to differentiate into macrophages, further perpetuating the inflammatory response and fostering fibrosis within the kidneys (25). The activation of monocytes and their intricate interactions with other immune cells are believed to propel the progression of DN. Results from single-cell sequencing conducted by Parker C. Wilson et al. (26) have revealed a notable increase in HLA-DR-marked monocytes in renal tissue samples obtained from early-stage DN patients. This finding aligns with our study’s observations that HLA-DR on CD14^+^ CD16^−^ monocytes and HLA-DR on CD14^+^ monocytes actively contribute to the development of DN. This suggests that HLA-DR may serve as a potential monitoring indicator or therapeutic target for DN. A study by Juan Jin et al. reported a negative correlation between HLA-DR-marked monocytes and the severity of DN (27), implying that additional research with a larger sample size may be warranted for a comprehensive understanding.

Dendritic cells play an indispensable role in both the genesis and advancement of DN. These cells instigate the immune response by presenting antigens to T cells, thereby triggering the production of pro-inflammatory cytokines and the recruitment of additional immune cells to the kidney (19). This orchestrated process contributes significantly to renal inflammation, extracellular matrix deposition, and the ensuing fibrosis. The activation and interplay of dendritic cells with other immune components in the kidney are believed to intricately contribute to the perpetuation of chronic inflammation and the progression of DN (28). Furthermore, our study outcomes underscore the influential role of HLA DR on Dendritic Cells and HLA DR on plasmacytoid Dendritic Cells in fostering the development of DN.

HLA-DR, a crucial molecule within the human leukocyte antigen system, belongs to the MHC-II class of molecules and comprises two major subunits: the α-subunit and the β-subunit, with molecular weights of 36 kD and 27 kD, respectively (29). It plays a pivotal role in the immune system. HLA-DR molecules are widely expressed on various immune cell surfaces, including B lymphocytes, monocytes, macrophages, activated T lymphocytes, activated NK lymphocytes, and human progenitor cells (30). This extensive expression pattern enables HLA-DR to interact with other immune cells during immune responses, thereby regulating and promoting the progression of these responses (31). HLA-DR plays a critical role in the recognition of foreign antigens during the activation of specific T cells in immune responses (32). It is closely associated with the expression of antigens on T cells during infection and the initiation of inflammatory cascade reactions. Therefore, the expression level of HLA-DR can serve as an important indicator reflecting the state of the immune system and the progression of diseases (33). Previous research has linked human leukocyte antigens (HLA) to renal function, yet there is conflicting evidence and limited agreement on the precise nature or significance of this relationship. Marcus Lowe (34) et al. found an association between certain haplotypes, including HLA-DRB103:01 and DQB102:01, and declining kidney function. In a comprehensive phenome-wide association study (PheWAS), the HLA-DRB104 and HLA-DQB103:02 alleles within the HLA-DR4-HLA-DQ8(3) risk haplotype showed associations with both T1DM and DKD (35). Our present comprehension of HLA mechanisms and disease advancement indicates that the impact of HLA on the individual predisposing conditions for kidney disease serves as the conduit for its role in advancing renal conditions. However, specific HLA variants could potentially stimulate a broader pro-fibrogenic T-cell phenotype, possibly exacerbating disease onset or progression (36).

While our study contributes valuable insights, it is essential to acknowledge its limitations. Firstly, depending on the degree of the disease, DN can be classified into different types; our study’s broad approach to DN types might dilute specific causal pathways pertinent to distinct DN categories. Secondly, when selecting instrumental variables, we set a threshold of 1 × 10^−5^, which increases the risk of pleiotropy of instrumental variables. Thirdly, the scope of our research was confined to individuals of European descent, and it is imperative to conduct additional investigations to discern the applicability of the results across diverse ethnic populations. At last, the sensitivity analysis revealed heterogeneity, suggesting potential variations in the findings. As a result, further clinical studies are imperative to corroborate and validate the outcomes of our research.

## Methods

### Ethics

The summary-level data of GWAS used in this study are publicly accessible, and the original study have acquired ethical approval and informed consent.

### Exposure of immunity-wide GWAS data sources

GWAS summary statistics for each immune trait are publicly available from the IEU GWAS (accession numbers from ebi-a-GCST90001391 to ebi-a-GCST90002121) (37). The original GWAS on immune traits was performed using data from in a cohort of 3,757 Sardinians after adjusting for covariates (i.e., sex, age). A total of 731 immunophenotypes including 118 absolute cell counts, 389 MFIs of surface antigens, and 32 morphological parameters were assessed by flow cytometry. We select the instrumental variables for immune cells by the following criteria: (1) SNPs at the genome-wide significance level (P<1×10^–5^); (2) SNP clumping using the PLINK algorithm (r^2^< 0.001, with a clumping window of 10,000 kb.) (23). We used F-statistic to verify the strength of IVs, which is calculated by the following formula: R^2^×(N−2)/(1−R^2^). We calculate R^2^ by the following formula: R^2^=[2× Beta^2^×(1−EAF)×EAF]/[2× Beta^2^× (1−EAF) × EAF+2 × SE^2^×N× (1−EAF) × EAF]. Specifically, Beta indicates the genetic effect of SNP on immune traits, EAF is the effect allele frequency, SE is the standard error, and N is the sample size. We only retained strong IVs (F-statistic>10) for each of the exposures (38) (**Supplementary Table 1**).

### Outcomes in GWAS: diabetic nephropathy phenotypes

Diabetic nephropathy as outcome was defined when there were glomerular disorders in the patients with diabetes mellitus with the criterion of ICD-10 (code: N08.3*). Patients meeting one of the following criteria are diagnosed with DN: (i) urinary albumin-to-creatinine ratio (ACR) greater than or equal to 30 mg/g (3.4 mg/mmol); (ii) urinary protein excretion rate greater than or equal to 30 mg/24 h; (iii) diabetes patients with a glomerular filtration rate (GFR) <60 mL/min/1.73 m² (39). The GWAS data correlated with diabetic nephropathy [IEU GWAS ID: finn-b-DM_NEPHROPATHY; N = 213746 (3,283 cases and 210,463 controls)] were obtained from the Integrative Epidemiologic Unit (IEU) GWAS database at https://gwas.mrcieu.ac.uk/. The total number of patients is 3,789, with 1,315 men and 2,474 women. The mean age at first event is 55.14 years (https://gwas.mrcieu.ac.uk/).

### Mendelian randomization analyses

We used five MR analytical methods which include standard inverse variance weighted (IVW), MR-Egger regression, weighted median, and simple mode to evaluate the causal effects of immune cells signature on DN. The Mendelian randomization pleiotropy residual sum was determined in this study. Moreover, the MR design must satisfy three key assumptions: (i) the genetic instruments reliably predict the exposure of interest (P < 1×10^–5^); (ii) the genetic instruments are independent of potential confounding factors; (iii) the genetic instruments influence the outcome solely through the identified risk factors. We chose standard inverse variance weighted (IVW) estimates as the main analysis. This method is a widely used method in Mendelian randomization (MR) analysis that combines estimates of causal effects from genetic variants by weighting them based on their inverse variances, providing a summary estimate of the overall causal effect (40). MR-Egger could evaluate whether genetic variants have pleiotropic effects on the outcome, as well as to estimate the causal effect (41). Weighted median MR uses most SNPs (majority of genetic variants) to determine the presence or absence of causality. Weighted-mode MR firstly groups SNPs into clusters and then calculates based on the cluster with the most SNPs (42). The simple mode approach involves clustering genetic variants and determining the causal effect based on the cluster with the greatest number of variants (43). This method offers a direct means to estimate the overall causal effect by making use of the majority of genetic variants. Furthermore, we performed reverse Mendelian randomization to analyze the causal effect of DN on the immune cell signatures.

### Sensitivity analysis

We used Cochran’s Q statistic, funnel pot, leave-one-out (LOO) analyses, and MR-Egger intercept tests to assess the pleiotropy. We calculate the P value of the Cochran Q test to test for heterogeneity. We also used the intercept term derived from MR-Egger regression to assess horizontal pleiotropy. LOO analysis could estimate whether the causal estimate was driven by any single SNP (44).

### Statistical analysis

For binary outcome, the MR estimates were presented as odds ratios (OR) with corresponding 95% confidence intervals (CI). All analyses were performed by the packages TwoSampleMR (version 0.4.25) in R (version 3.6.1). The p-values were adjusted using the Benjamini & Hochberg (BH) method, and those with adjusted p-values less than 0.05 were considered to have significant differences. Moreover, we used an online power calculation tool for MR (https://shiny.cnsgenomics.com/mRnd/) to calculate the statistical power of causal effect estimates (45). A power threshold of 0.8 was considered appropriate, enabling the rejection of 4/5 false null hypotheses (46). The STROBE-MR checklist of recommended items to address in reports of Mendelian randomization studies is presented in **Supplementary Table 7**.

## Data Availability

The original contributions presented in the study are included in the article/**Supplementary Material**. Further inquiries can be directed to the corresponding authors.
