# Supplementary figures and images for: Causal role of immune cells in diabetic nephropathy: a bidirectional Mendelian randomization study
Source: Front Endocrinol (Lausanne). 2024 Sep 13;15:1357642. doi: 10.3389/fendo.2024.1357642 (PMC11427287; doi:10.3389/fendo.2024.1357642)

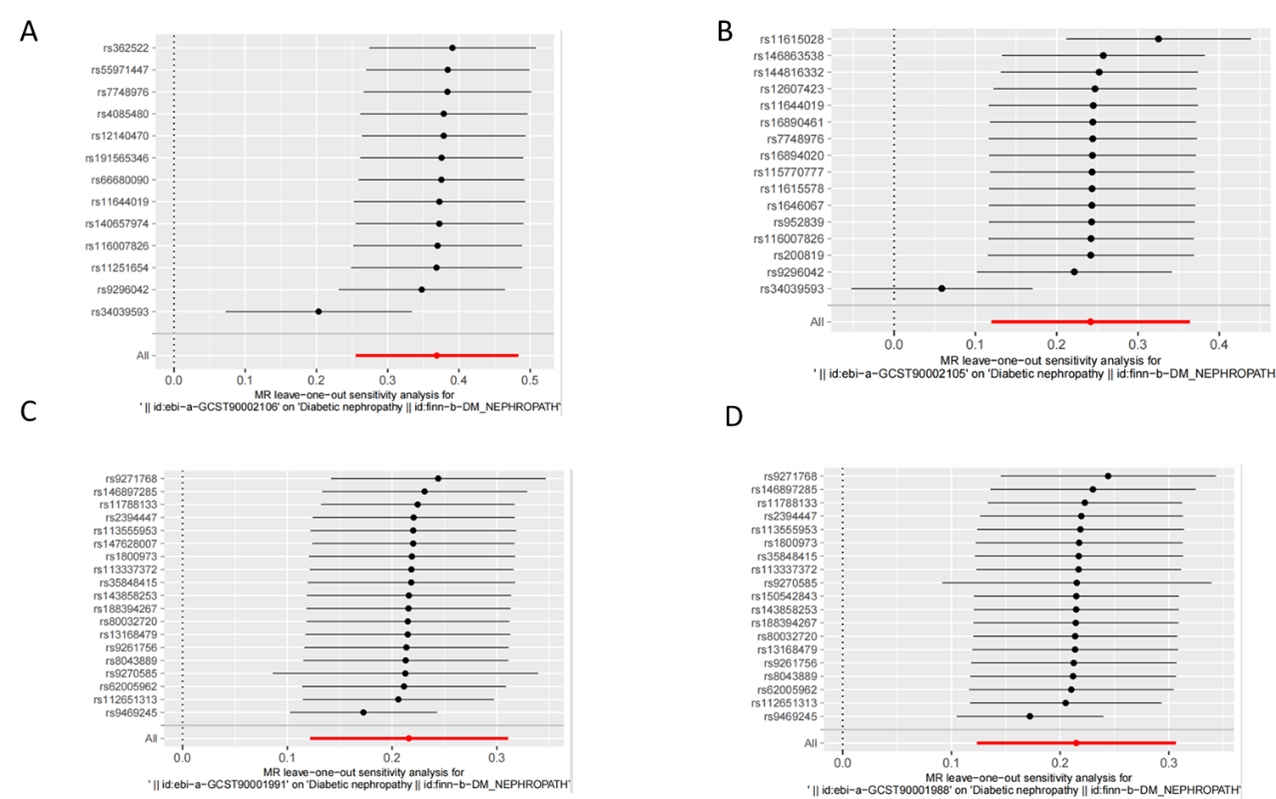

Supplement: Supplementary file 1 [file Image1.tif]
