# Supplementary material for: Causal role of immune cells in diabetic nephropathy: a bidirectional Mendelian randomization study
Source: Front Endocrinol (Lausanne). 2024 Sep 13;15:1357642. doi: 10.3389/fendo.2024.1357642 (PMC11427287; doi:10.3389/fendo.2024.1357642)
Supplement: Supplementary file 8 [file Table7.docx]

**STROBE-MR checklist of recommended items to address in reports of Mendelian randomization studies**^1^ ^2^

| **Item No.** | **Section** | **Checklist item** | **Relevant text from manuscript** |
| --- | --- | --- | --- |
| 1 | **TITLE and ABSTRACT** | Indicate Mendelian randomization (MR) as the study’s design in the title and/or the abstract if that is a main purpose of the study | Causal Role of Immune Cells in Diabetic Nephropathy: A Bidirectional Mendelian randomization (MR) study |
|  | **INTRODUCTION** |  |  |
| 2 | **Background** | Explain the scientific background and rationale for the reported study. What is the exposure? Is a potential causal relationship between exposure and outcome plausible? Justify why MR is a helpful method to address the study question | we employed Mendelian randomization (MR) analysis to examine the causal associations between 731 immune cell signatures and the risk of DN. Current research indicates that DN is an inflammatory illness, and immune cells from both innate and adaptive immunity, including macrophages and T cells, may contribute to the illness's exacerbation. Mendelian randomization study is a statistical method that can unveil causal relationships. |
| 3 | **Objectives** | State specific objectives clearly, including pre-specified causal hypotheses (if any). State that MR is a method that, under specific assumptions, intends to estimate causal effects | We assume a causal relationship between immune cell features and the onset of diabetic nephropathy. |
|  | **METHODS** |  |  |
| 4 | **Study design and data sources** | Present key elements of the study design early in the article. Consider including a table listing sources of data for all phases of the study. For each data source contributing to the analysis, describe the following: |  |
|  | a) | Setting: Describe the study design and the underlying population, if possible. Describe the setting, locations, and relevant dates, including periods of recruitment, exposure, follow-up, and data collection, when available. | The original GWAS on immune traits was performed using data from in a cohort of 3,757 Sardinians after adjusting for covariates (i.e., sex, age and age). The GWAS data correlated with diabetic nephropathy [IEU GWAS ID: finn-b-DM_NEPHROPATHY; N = 213746 (3,283 cases and 210,463 controls)] were obtained from the Integrative Epidemiologic Unit (IEU) GWAS database at https://gwas.mrcieu.ac.uk/. |
|  | b) | Participants: Give the eligibility criteria, and the sources and methods of selection of participants. Report the sample size, and whether any power or sample size calculations were carried out prior to the main analysis | We used F-statistic to verify the strength of IVs, which is calculated by the following formula: R2×(N−2)/(1−R2). We calculate R2 by the following formula: R2=[2× Beta2×(1−EAF)×EAF]/[2× Beta2× (1−EAF) × EAF+2 × SE2×N× (1−EAF) × EAF]. |
|  | c) | Describe measurement, quality control and selection of genetic variants | We select the instrumental variables for immune cells by the following criteria: (1) SNPs at the genome-wide significance level (P<1×10–5); (2) SNP clumping using the PLINK algorithm (r2< 0.001, with a clumping window of 10000 kb.) |
|  | d) | For each exposure, outcome, and other relevant variables, describe methods of assessment and diagnostic criteria for diseases | Diabetic nephropathy as outcome was defined when there was glomerular disorders in the patients with diabetes mellitus with the criterion of ICD-10 (code: N08.3*). |
|  | e) | Provide details of ethics committee approval and participant informed consent, if relevant | As MR study utilizes summary statistics from previously published GWAS studies, an ethics statement may not be necessary for this study. |
| 5 | **Assumptions** | Explicitly state the three core IV assumptions for the main analysis (relevance, independence and exclusion restriction) as well assumptions for any additional or sensitivity analysis | Moreover, the MR design must satisfy three key assumptions: (i) the genetic instruments reliably predict the exposure of interest (P < 5 × 10–8); (ii) the genetic instruments are independent of potential confounding factors; (iii) the genetic instruments influence the outcome solely through the identified risk factors. |
| 6 | **Statistical methods: main analysis** | Describe statistical methods and statistics used |  |
|  | a) | Describe how quantitative variables were handled in the analyses (i.e., scale, units, model) | For binary outcome, the MR estimates were presented as odds ratios (OR) with corresponding 95% confidence intervals (CI). |
|  | b) | Describe how genetic variants were handled in the analyses and, if applicable, how their weights were selected | We used F-statistic to verify the strength of IVs, which is calculated by the following formula: R2×(N−2)/(1−R2). We calculate R2 by the following formula: R2=[2× Beta2×(1−EAF)×EAF]/[2× Beta2× (1−EAF) × EAF+2 × SE2×N× (1−EAF) × EAF]. |
|  | c) | Describe the MR estimator (e.g. two-stage least squares, Wald ratio) and related statistics. Detail the included covariates and, in case of two-sample MR, whether the same covariate set was used for adjustment in the two samples | We used five MR analytical methods which include standard inverse variance weighted (IVW) 、MR-Egger regression, weighted median, simple mode to evaluate the causal effects of immune cells signature on DN. |
|  | d) | Explain how missing data were addressed | No missing data |
|  | e) | If applicable, indicate how multiple testing was addressed |  |
| 7 | **Assessment of assumptions** | Describe any methods or prior knowledge used to assess the assumptions or justify their validity | We chose standard inverse variance weighted (IVW) estimates as the main analysis. This method is a widely used method in Mendelian randomization (MR) analysis that combines estimates of causal effects from genetic variants by weighting them based on their inverse variances, providing a summary estimate of the overall causal effect[16]. MR-Egger could evaluate whether genetic variants have pleiotropic effects on the outcome, as well as to estimate the causal effect[17]. Weighted median MR uses most SNPs (majority of genetic variants) to determine the presence or absence of causality. Weighted-mode MR firstly groups SNPs into clusters, and then calculates based on the cluster with the most SNPs[18]. The simple mode approach involves clustering genetic variants and determining the causal effect based on the cluster with the greatest number of variants[19]. This method offers a direct means to estimate the overall causal effect by making use of the majority of genetic variants. |
| 8 | **Sensitivity analyses and additional analyses** | Describe any sensitivity analyses or additional analyses performed (e.g. comparison of effect estimates from different approaches, independent replication, bias analytic techniques, validation of instruments, simulations) | We used Cochran’s Q statistic, funnel pot, leave-one-out (LOO) analyses and MR-Egger intercept tests to assess the pleiotropy. We calculate the P value of the Cochran Q test to test for heterogeneity. And we also used intercept term derived from MR-Egger regression to assess horizontal pleiotropy. LOO analysis could estimate whether the causal estimate was driven by any single SNP[20]. |
| 9 | **Software and pre-registration** |  |  |
|  | a) | Name statistical software and package(s), including version and settings used | All analyses were performed by the packages TwoSampleMR (version 0.4.25) in R (version 3.6.1). |
|  | b) | State whether the study protocol and details were pre-registered (as well as when and where) | No pre-registered. |
|  | **RESULTS** |  |  |
| 10 | **Descriptive data** |  |  |
|  | a) | Report the numbers of individuals at each stage of included studies and reasons for exclusion. Consider use of a flow diagram | Figure 1. Description of the study design in this bidirectional MR study. |
|  | b) | Report summary statistics for phenotypic exposure(s), outcome(s), and other relevant variables (e.g. means, SDs, proportions) | We performed a Mendelian randomization analysis as shown in Figure 1. After BH adjustment (P.adjusted<0.05), we detected four immunophenotypes which have promoted effects on DN: HLA DR on Dendritic Cell (dendritic cell panel), HLA DR on CD14+ CD16- monocyte (monocyte panel), HLA DR on CD14+ monocyte (monocyte panel) and HLA DR on plasmacytoid Dendritic Cell (dendritic cell panel). The odds ratio (OR) of HLA DR on Dendritic Cell on diabetic nephropathy risk was estimated to be 1.4460 (95% CI= 1.2904~1.6205, P=2.18×10−10, P.adjusted= 1.6×10−7，Figure 2) by using the IVW method. The OR of HLA DR on CD14+ CD16- monocyte on DN risk was estimated to be 1.2396(95% CI=1.1315~1.3580, P=3.93×10−6, P.adjusted = 0.0014, Figure 2) by using the IVW method. The OR of HLA DR on CD14+ monocyte on DN risk was estimated to be 1.2411 (95% CI=1.1295~1.3637, P=6.97×10-6, P.adjusted=0.0016, Figure 2) by using the IVW method. The OR of HLA DR on plasmacytoid Dendritic Cell on DN risk was estimated to be 1.2733 (95% CI= 1.1273~ 1.4382, P= 0.0001, P.adjusted = 0.01835, Figure 2, Supplementary Table2) by using the IVW method. Similar results were obtained using four additional methods (Supplementary Table3). |
|  | c) | If the data sources include meta-analyses of previous studies, provide the assessments of heterogeneity across these studies | No meta-analysis |
|  | d) | For two-sample MR:  i.  Provide justification of the similarity of the genetic variant-exposure associations between the exposure and outcome samples  ii.  Provide information on the number of individuals who overlap between the exposure and outcome studies | Since exposure and outcomes are derived from different database cohorts, there is no population overlap. |
| 11 | **Main results** |  |  |
|  | a) | Report the associations between genetic variant and exposure, and between genetic variant and outcome, preferably on an interpretable scale | We performed a Mendelian randomization analysis as shown in Figure 1. After BH adjustment (P.adjusted<0.05), we detected four immunophenotypes which have promoted effects on DN: HLA DR on Dendritic Cell (dendritic cell panel), HLA DR on CD14+ CD16- monocyte (monocyte panel), HLA DR on CD14+ monocyte (monocyte panel) and HLA DR on plasmacytoid Dendritic Cell (dendritic cell panel). |
|  | b) | Report MR estimates of the relationship between exposure and outcome, and the measures of uncertainty from the MR analysis, on an interpretable scale, such as odds ratio or relative risk per SD difference | The odds ratio (OR) of HLA DR on Dendritic Cell on diabetic nephropathy risk was estimated to be 1.4460 (95% CI= 1.2904~1.6205, P=2.18×10−10, P.adjusted= 1.6×10−7，Figure 2) by using the IVW method. The OR of HLA DR on CD14+ CD16- monocyte on DN risk was estimated to be 1.2396(95% CI=1.1315~1.3580, P=3.93×10−6, P.adjusted = 0.0014, Figure 2) by using the IVW method. The OR of HLA DR on CD14+ monocyte on DN risk was estimated to be 1.2411 (95% CI=1.1295~1.3637, P=6.97×10-6, P.adjusted=0.0016, Figure 2) by using the IVW method. The OR of HLA DR on plasmacytoid Dendritic Cell on DN risk was estimated to be 1.2733 (95% CI= 1.1273~ 1.4382, P= 0.0001, P.adjusted = 0.01835, Figure 2, Supplementary Table2) by using the IVW method. Similar results were obtained using four additional methods (Supplementary Table3). Figure 3 showed the scatter plot for effect sizes of SNPs for immunophenotypes and those for DN. |
|  | c) | If relevant, consider translating estimates of relative risk into absolute risk for a meaningful time period |  |
|  | d) | Consider plots to visualize results (e.g. forest plot, scatterplot of associations between genetic variants and outcome versus between genetic variants and exposure) | Figure 2. Causal effects for immune traits on DN susceptibility. |
| 12 | **Assessment of assumptions** |  | The p-values were adjusted using the Benjamini & Hochberg（BH）method, and those with adjusted p-values less than 0.05 were considered to have significant differences. |
|  | a) | Report the assessment of the validity of the assumptions |  |
|  | b) | Report any additional statistics (e.g., assessments of heterogeneity across genetic variants, such as *I^2^*, Q statistic or E-value) |  |
| 13 | **Sensitivity analyses and additional analyses** |  |  |
|  | a) | Report any sensitivity analyses to assess the robustness of the main results to violations of the assumptions | MR-Egger intercept(P>0.05) indicated that only HLA DR on Dendritic Cell demonstrated pleiotropy in our results (Table 1, Supplementary Table4). Furthermore, the results in Table 1 and Supplementary Table5 showed that all P values of Q test analysis were<0.05, indicating that heterogeneity existed. Importantly, As shown in the leave-one-out analysis results, no marked difference was found in causal estimations of immune cell signatures on DN, suggesting that none of the identified causal associations were driven by any single IV (Supplementary Figure 1). |
|  | b) | Report results from other sensitivity analyses or additional analyses | Similar results were obtained using four additional methods (Supplementary Table3). |
|  | c) | Report any assessment of direction of causal relationship (e.g., bidirectional MR) | The reverse Mendelian randomization study did not find any causal relationship between diabetic nephropathy and the four immune cell types we identified（Table 2）. Furthermore, we used diabetic nephropathy as the exposure and 731 immune cell types as the outcome, and the results showed that none of the p-values passed multiple testing corrections (Supplementary Table 6). |
|  | d) | When relevant, report and compare with estimates from non-MR analyses | Results from single-cell sequencing conducted by Parker C. Wilson et al. [9] have revealed a notable increase in HLA-DR-marked monocytes in renal tissue samples obtained from early-stage diabetic nephropathy patients. This finding aligns with our study's observations that HLA-DR on CD14+ CD16- monocytes and HLA-DR on CD14+ monocytes actively contribute to the development of diabetic nephropathy. This suggests that HLA-DR may serve as a potential monitoring indicator or therapeutic target for diabetic nephropathy. However, a study by Juan Jin et al. reported a negative correlation between HLA-DR-marked monocytes and the severity of diabetic nephropathy [10], implying that additional research with a larger sample size may be warranted for a comprehensive understanding. |
|  | e) | Consider additional plots to visualize results (e.g., leave-one-out analyses) | Scatter plots from genetically predicted immunophenotypes on DN. |
|  | **DISCUSSION** |  |  |
| 14 | **Key results** | Summarize key results with reference to study objectives | This study marks a pioneering use of Mendelian randomization to scrutinize the causal connection between immune cell characteristics and the susceptibility to diabetic nephropathy. The findings unequivocally indicate that only four distinct immune cell types exhibit a causal association with the risk of diabetic nephropathy. |
| 15 | **Limitations** | Discuss limitations of the study, taking into account the validity of the IV assumptions, other sources of potential bias, and imprecision. Discuss both direction and magnitude of any potential bias and any efforts to address them | While our study contributes valuable insights, it is essential to acknowledge its limitations. Firstly, the scope of our research was confined to individuals of European descent, and it is imperative to conduct additional investigations to discern the applicability of the results across diverse ethnic populations. Secondly, the sensitivity analysis revealed heterogeneity, suggesting potential variations in the findings. As a result, further clinical studies are imperative to corroborate and validate the outcomes of our research. |
| 16 | **Interpretation** |  |  |
|  | a) | Meaning: Give a cautious overall interpretation of results in the context of their limitations and in comparison with other studies | This study marks a pioneering use of Mendelian randomization to scrutinize the causal connection between immune cell characteristics and the susceptibility to diabetic nephropathy. The findings indicate that only four distinct immune cell types exhibit a causal association with the risk of diabetic nephropathy. |
|  | b) | Mechanism: Discuss underlying biological mechanisms that could drive a potential causal relationship between the investigated exposure and the outcome, and whether the gene-environment equivalence assumption is reasonable. Use causal language carefully, clarifying that IV estimates may provide causal effects only under certain assumptions | Monocytes play a pivotal role in the pathogenesis of diabetic nephropathy[7]. Heightened infiltration of monocytes into renal tissue has been documented in patients with diabetic nephropathy, where these cells contribute to the release of pro-inflammatory cytokines, including tumor necrosis factor-alpha (TNF-α) and interleukin-1 beta (IL-1β), thereby initiating renal inflammation and subsequent tissue damage. Moreover, monocytes have the ability to differentiate into macrophages, further perpetuating the inflammatory response and fostering fibrosis within the kidneys[8]. The activation of monocytes and their intricate interactions with other immune cells are believed to propel the progression of diabetic nephropathy. |
|  | c) | Clinical relevance: Discuss whether the results have clinical or public policy relevance, and to what extent they inform effect sizes of possible interventions | A study by Juan Jin et al. reported a negative correlation between HLA-DR-marked monocytes and the severity of diabetic nephropathy [10], implying that additional research with a larger sample size may be warranted for a comprehensive understanding.  The activation and interplay of dendritic cells with other immune components in the kidney are believed to intricately contribute to the perpetuation of chronic inflammation and the progression of diabetic nephropathy |
| 17 | **Generalizability** | Discuss the generalizability of the study results (a) to other populations, (b) across other exposure periods/timings, and (c) across other levels of exposure | This study marks a pioneering use of Mendelian randomization to scrutinize the causal connection between immune cell characteristics and the susceptibility to diabetic nephropathy. The findings indicate that only four distinct immune cell types exhibit a causal association with the risk of diabetic nephropathy. |
|  | **OTHER INFORMATION** |  |  |
| 18 | **Funding** | Describe sources of funding and the role of funders in the present study and, if applicable, sources of funding for the databases and original study or studies on which the present study is based | This work was supported by National Natural Science Foundation of China (82072207, 81971861); and Shanghai Municipal Science and Technology Commission Shanghai Excellent Discipline Leader Program (21XD1402200). |
| 19 | **Data and data sharing** | Provide the data used to perform all analyses or report where and how the data can be accessed, and reference these sources in the article. Provide the statistical code needed to reproduce the results in the article, or report whether the code is publicly accessible and if so, where | GWAS summary statistics are publicly available from the IEU GWAS. |
| 20 | **Conflicts of Interest** | All authors should declare all potential conflicts of interest | The authors declare that they have no competing interests. |

This checklist is copyrighted by the Equator Network under the Creative Commons Attribution 3.0 Unported (CC BY 3.0) license.

1. Skrivankova VW, Richmond RC, Woolf BAR, Yarmolinsky J, Davies NM, Swanson SA, et al. Strengthening the Reporting of Observational Studies in Epidemiology using Mendelian Randomization (STROBE-MR) Statement. JAMA. 2021;under review.

2. Skrivankova VW, Richmond RC, Woolf BAR, Davies NM, Swanson SA, VanderWeele TJ, et al. Strengthening the Reporting of Observational Studies in Epidemiology using Mendelian Randomisation (STROBE-MR): Explanation and Elaboration. BMJ. 2021;375:n2233.
